# Multi-level profiling unravels mitochondrial dysfunction in myotonic dystrophy type 2

**DOI:** 10.1007/s00401-023-02673-y

**Published:** 2024-01-19

**Authors:** Felix Kleefeld, Rita Horvath, Iago Pinal-Fernandez, Andrew L. Mammen, Maria Casal-Dominguez, Denisa Hathazi, Sarah Melchert, Katrin Hahn, Albert Sickmann, Claudia Muselmann-Genschow, Andreas Hentschel, Corinna Preuße, Andreas Roos, Benedikt Schoser, Werner Stenzel

**Affiliations:** 1 Department of Neurology, Charité-Universitätsmedizin Berlin, Corporate Member of Freie Universität Berlin, Humboldt-Universität Zu Berlin, Berlin Institute of Health (BIH), Charitéplatz 1, 10117 Berlin, Germany; 2 Department of Clinical Neurosciences, University of Cambridge, Cambridge, UK; 3 Muscle Disease Unit, National Institute of Arthritis and Musculoskeletal and Skin Diseases, National Institutes of Health, Bethesda, MD 20892 USA; 4 Department of Neurology, Johns Hopkins University School of Medicine, Baltimore, MD 21205 USA; 5 Department of Neurology, Charité-Universitätsmedizin Berlin, Corporate Member of Freie Universität Berlin, Humboldt-Universität Zu Berlin, Charitéplatz 1, 10117 Berlin, Germany; 6 Leibniz-Institut Für Analytische Wissenschaften-ISAS E.V., 44139 Dortmund, Germany; 7 Department of Neuropathology, Charité-Universitätsmedizin Berlin, Corporate Member of Freie Universität Berlin, Humboldt-Universität Zu Berlin, Berlin Institute of Health (BIH), Charitéplatz 1, 10117 Berlin, Germany; 8 Department of Neuropediatrics, Charité-Universitätsmedizin Berlin, Corporate Member of Freie Universität Berlin, Humboldt-Universität Zu Berlin, Berlin Institute of Health (BIH), Augustenburger Platz 1, 13353 Berlin, Germany; 9 Pediatric Neurology, Faculty of Medicine, University Children’s Hospital, University of Duisburg-Essen, Essen, Germany; 10 Children’s Hospital of Eastern Ontario Research Institute, University of Ottawa, Ottawa, ON K1H 8L1 Canada; 11 Department of Neurology, Friedrich-Baur-Institute, Ludwig-Maximilians-University, Munich, Germany

**Keywords:** Myotonic dystrophy type 2, Proximal myotonic myopathy, Mitochondrial dysfunction

## Abstract

Myotonic dystrophy type 2 (DM2) is an autosomal-dominant multisystemic disease with a core manifestation of proximal muscle weakness, muscle atrophy, myotonia, and myalgia. The disease-causing CCTG tetranucleotide expansion within the *CNBP* gene on chromosome 3 leads to an RNA-dominated spliceopathy, which is currently untreatable. Research exploring the pathophysiological mechanisms in myotonic dystrophy type 1 has resulted in new insights into disease mechanisms and identified mitochondrial dysfunction as a promising therapeutic target. It remains unclear whether similar mechanisms underlie DM2 and, if so, whether these might also serve as potential therapeutic targets. In this cross-sectional study, we studied DM2 skeletal muscle biopsy specimens on proteomic, molecular, and morphological, including ultrastructural levels in two separate patient cohorts consisting of 8 (explorative cohort) and 40 (confirmatory cohort) patients. Seven muscle biopsy specimens from four female and three male DM2 patients underwent proteomic analysis and respiratory chain enzymology. We performed bulk RNA sequencing, immunoblotting of respiratory chain complexes, mitochondrial DNA copy number determination, and long-range PCR (LR-PCR) to study mitochondrial DNA deletions on six biopsies. Proteomic and transcriptomic analyses revealed a downregulation of essential mitochondrial proteins and their respective RNA transcripts, namely of subunits of respiratory chain complexes I, III, and IV (e.g., mt-CO1, mt-ND1, mt-CYB, NDUFB6) and associated translation factors (TACO1). Light microscopy showed mitochondrial abnormalities (e.g., an age-inappropriate amount of COX-deficient fibers, subsarcolemmal accumulation) in most biopsy specimens. Electron microscopy revealed widespread ultrastructural mitochondrial abnormalities, including dysmorphic mitochondria with paracrystalline inclusions. Immunofluorescence studies with co-localization of autophagy (p62, LC-3) and mitochondrial marker proteins (TOM20, COX-IV), as well as immunohistochemistry for mitophagy marker BNIP3 indicated impaired mitophagic flux. Immunoblotting and LR-PCR did not reveal significant differences between patients and controls. In contrast, mtDNA copy number measurement showed a reduction of mtDNA copy numbers in the patient group compared to controls. This first multi-level study of DM2 unravels thus far undescribed functional and structural mitochondrial abnormalities. However, the molecular link between the tetranucleotide expansion and mitochondrial dysfunction needs to be further elucidated.

**Supplementary Information:**

The online version contains supplementary material available at 10.1007/s00401-023-02673-y.

## Introduction

Myotonic dystrophy type 2 (DM2; synonym: proximal myotonic myopathy, PROMM) is an autosomal-dominant, multisystemic disease-causing, among other symptoms, proximal muscle weakness and atrophy and myalgia. An RNA-dominated spliceopathy causes the multisystem organ vulnerability of DM2 due to CCTG tetranucleotide expansions within the *CNBP* gene (located on chromosome 3). Clinical hallmark features include cataracts, cardiac abnormalities, and glucose intolerance [13]. Although DM2 is considered to be less prevalent than myotonic dystrophy type 1 (DM1) [10], it is one of the most common adult muscular diseases in Central and North-Eastern Europe [23]. Based on the heterogeneity of symptoms and the genetic background, DM2 is assumed to be highly under- and misdiagnosed, leading to suboptimal management of patients. Research on myotonic dystrophies has focused on DM1, with biomarker discovery and molecular studies [5, 11], showing promising results and opening windows toward therapeutic trials. Beyond the key therapeutic target aiming to correct spliceopathy, metabolic abnormalities, including respiratory chain dysfunction, have been identified as potential treatment targets in DM1 [5]. In addition, biomarker candidates such as cell-free RNA have emerged [11, 14]. In DM2, however, comparable molecular studies and reliable biomarkers predicting disease progression and severity are lacking. It remains in part unclear whether similar pathophysiological mechanisms are involved in both types of myotonic dystrophy and whether similar contributors to molecular pathology (e.g., spliceopathy and RNA or mitochondrial metabolism) may be identified as alternative treatment targets or biomarkers. Therefore, a better understanding of the molecular pathogenesis of DM2 is paramount to advance trial readiness and the development of therapeutic strategies.

This study used a multi-level approach to study histomorphological, ultrastructural, proteomic, and transcriptomic data on two independent German cohorts of DM2 patients. Here, we report and correlate proteomic, transcriptomic and morphological data obtained from skeletal muscle biopsy specimens, identifying unreported mitochondrial abnormalities in skeletal muscles from DM2 patients. These abnormalities affect respiratory chain complexes I, III and IV and an associated translation factor (TACO1) and manifest on biochemical, morphological, and ultrastructural levels. Hence, our data point toward mitochondrial dysfunction being an essential factor in the pathogenesis of DM2. The molecular link, however, between mitochondrial dysfunction, the disease-causing tetranucleotide expansion in the *CNBP* gene, and cellular downstream effects on the RNA and protein level, needs further elucidation.

## Materials and methods

The experiments for this multi-center study were conducted on two independent DM2 cohorts from Berlin, Germany (Charité—Universitätsmedizin Berlin, Dep. of Neuropathology; histology and electron microscopy), and Munich, Germany (Friedrich-Baur-Institute, Dep. of Neurology; histology, electron microscopy and respiratory chain enzymology). Additional analyses were conducted in Cambridge, UK (University of Cambridge, Dep. of Clinical Neurosciences; immunoblotting, long-range and qPCR), Dortmund, Germany (Leibniz-Institut für Analytische Wissenschaften ISAS e.V.; proteomic profiling studies) and Bethesda, MD, USA (National Institutes of Health; bulk RNA sequencing). The study was approved by the institutional Ethics Review Board of the Charité—Universitätsmedizin Berlin (EA2/107/14; EA1/019/22), and a Material Transfer Agreement (MTA) was established between the institutions. The study was undertaken in accordance with the 1964 Declaration of Helsinki. Patient and control data were anonymized by the policies of the local institutional ethics review board.

### Patient cohort

All patients included in this study were recruited at the neuromuscular outpatient clinics at Charité, Berlin and Friedrich-Baur-Institute, Munich. The inclusion criteria were age > 18 years and positive genetic testing for myotonic dystrophy type 2 (*CNBP* repeat length > 75). All patients gave written informed consent to participate in the study. Within the large patient cohort of genetically confirmed DM2, we identified eight patients (four male, four female) who recently underwent a muscle biopsy for diagnostic purposes. Light and electron microscopy findings in these patients (exploratory cohort) were confirmed in an independent (confirmatory) second DM2 cohort (*n* = 40) from Munich, Germany, that included cases collected between 1985 and 2022. The workflow and cohorts are illustrated in Supplemental Fig. 1.

**Fig. 1:**
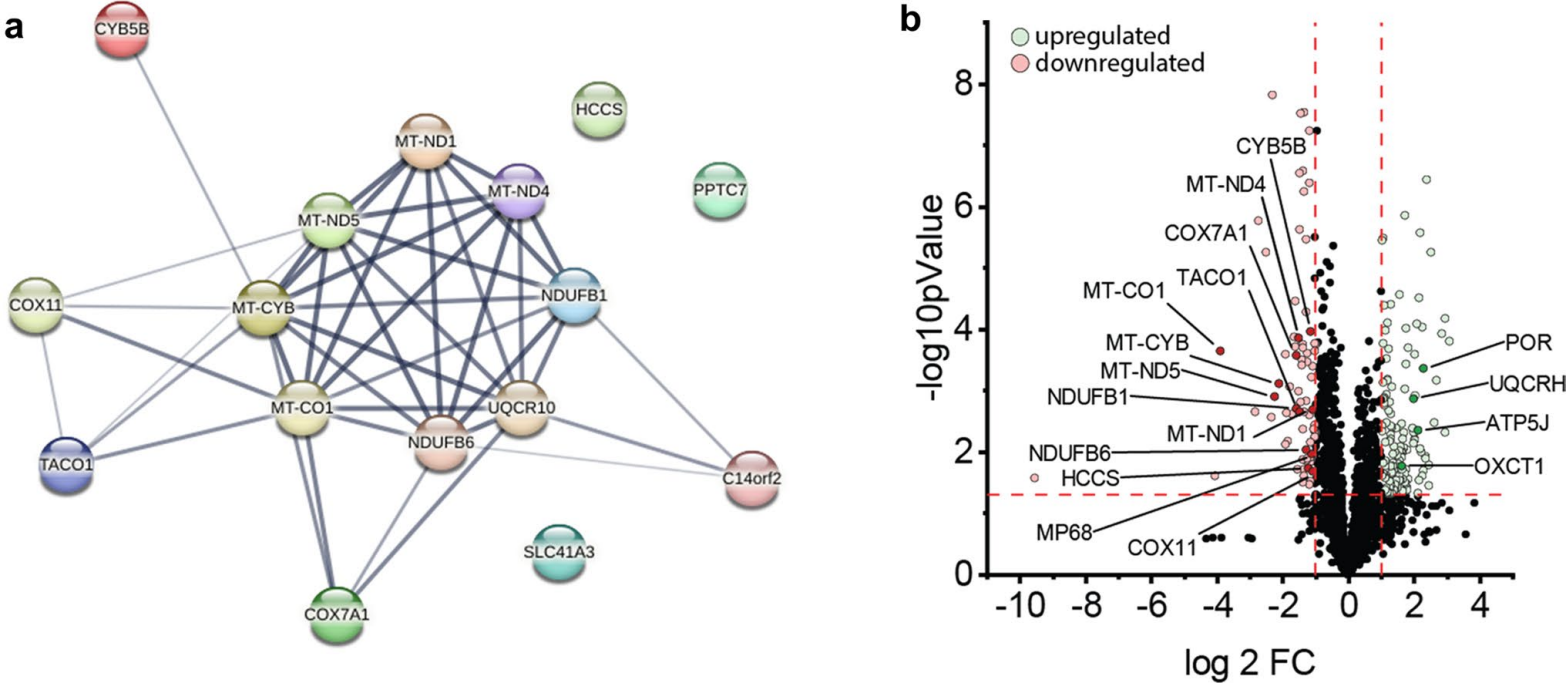
Proteomic studies performed on DM2-derived skeletal muscle biopsy samples. **a** STRING analysis of highly downregulated mitochondrial proteins illustrating the strong molecular interaction network, mainly affecting proteins related to OXPHOS complex I, III and IV assembly. **b** Volcano plot illustrating up- and downregulated proteins. Up- (green) and downregulated (red) mitochondria-associated proteins are highlighted

### Histological and (immuno-)histochemical analysis

We analyzed skeletal muscle biopsy specimens from 48 DM2 patients and 10 non-diseased controls (NDC). All biopsies were collected from the quadriceps femoris muscle. Before analysis, all skeletal muscle specimens had been cryopreserved at − 80 °C or fixed in glutaraldehyde (GA). In the exploratory cohort, the full spectrum of histopathological stains was performed. In the confirmatory cohort, fresh-frozen material was not available for additional stains from older biopsies, but at least one enzyme histochemical preparation for mitochondrial respiratory chain enzymes (COX–SDH, SDH, COX, NADH-TR) was available for each specimen.

Muscle biopsy samples were analyzed following standardized procedures [18, 19]. Routine stains, enzyme histochemistry, immunohistochemical, and double immunofluorescence reactions were carried out as described previously by [18, 19]. In brief, 7-µm-thick cryosections were stained with hematoxylin and eosin, modified Gömöri trichrome, COX–SDH enzyme histochemistry, NADH-TR, for nonspecific esterase, acid phosphatase, and other standard stains as described in a consensus statement [25]. Microscopic analyses were conducted by three myopathologists (FK, BS, WS).

### Transmission electron microscopy

For transmission electron microscopy (TEM), muscle biopsy specimens were fixed and embedded according to standard protocols. In brief, muscle specimens were fixed in 2.5% GA diluted in 0.1 M sodium cacodylate buffer for a minimum of 24 h at four ℃, osmicated in 1% osmium tetroxide in 0.05 M sodium cacodylate buffer, dehydrated using graded acetone series including combined en bloc staining with 1% uranyl acetate and 0.1% phosphotungstic acid in 70% acetone, infiltrated in RenLam resin, and then polymerized for 48–72 h at 60 ℃. Semithin Sects. (500 nm) were stained with Richardson solution (methylene blue) for microanatomical examination, and ultrathin Sects. (60–70 nm) were stained with uranyl acetate and lead citrate. Next, conventional ultrastructural analysis was performed using TEM 902 and TEM 906 (Zeiss, Oberkochen, Germany).

### Proteomic studies

Muscle biopsy specimens were prepared for proteomic analysis as described previously [9]. The analysis included five muscle biopsy samples from five adult male and two female patients with an unremarkable family history, normal histological studies, and normal laboratory workup, hence serving as non-diseased controls (NDC). These individuals had been biopsied diagnostically for muscle pain but revealed no pathological changes based on intensive microscopic workup. In brief, we subjected the samples to a bottom-up unbiased proteomic approach with label-free peptide quantification according to our published data-independent-acquisition (DIA) workflow [10]. For further downstream analysis, we considered only proteins identified with a minimum of > 2 unique peptides in the patient and control groups.

### Quantification of mitochondrial DNA copy number (mtDNA-CN)

The relative mtDNA copy number per cell was quantified by a multiplex Taqman qPCR assay (Bio-Rad 4,369,510), following a previously published protocol [8]. *B2M* transcript levels were used as nuclear-encoded reference gene/control, and *MT-ND1* was used as mitochondrial-encoded reference gene/control. The primers used for the qPCR reaction were as follows: *B2M* Fw-CACTGAAAAAGATGAGTATGCC, Rv-AACATTCCCTGACAATCCC; *MT-ND1* Fw-ACGCCATAAAACTCTTCACCAAAG, Rv-GGGTTCATAGTAGAAGAGCGATGG. All samples were run in triplicates and replicates greater than 0.5 Ct difference were removed. The relative amount of mtDNA was calculated at the Ct value difference of *B2M* and *MT-ND1*, where Delta *C*_t_ (ΔC_*t*_) equals the sample C_*t*_ of the mitochondrial gene (*MT-ND1*) subtracted from the sample C_*t*_ of the nuclear reference gene (*B2M*). For this analysis, six healthy and age-matched controls (four female, two male) were included. Control individuals had undergone a muscle biopsy for nonspecific muscle pain, showing normal muscle histology and complete clinical and laboratory workup.

### Respiratory chain immunoblotting and enzymology

Muscle biopsy samples were lysed in 100 μl of lysis buffer (50 mM Tris–HCl- Applichem Biochemica A3452 (pH 7.8) 150 mM NaCl (Merck 1,064,041,000), 1% SDS (Carl Roth CN30.1) and one tablet Complete Mini (Roche, 11,873,580,001) using a manual glass grinder followed by sonication. Samples were centrifuged for 5 min at four ℃ and 5000 × *g*. The protein concentration of the supernatant was determined with the Pierce™ Rapid Gold BCA Protein-Assay-Kit (Thermo Fisher A53225, according to the manufacturer’s protocol).

For immunoblotting, 10 μg of total protein extract was used for each sample analyzed. The samples were loaded on a gradient polyacrylamide gel (NuPage 4–12% Bis–Tris Protein gel, Thermo Fisher WG1402BOX, NP0321BOX) and separated for 60 min at 150 V. Proteins were then transferred to a PVDF membrane (Invitrogen transfer stacks IB24002) using the iBlot2 dry transfer system (Thermo Fisher IB21001) according to the manufacturer’s protocol. Membranes were blocked with 5% milk powder diluted in 1 × PBS-T (PBS: Gibco 18,912,014; 0.1% Tween-20: Sigma-Aldrich P7949) for 2 h and four washing steps using PBS-T. Membranes were next incubated with primary antibodies (Total OXPHOS Human WB Antibody Cocktail, Abcam, ab110411; dilution 1:800; TOM20: Recombinant Anti-TOM20 antibody, ab186735, dilution 1:2,000; GAPDH: anti-GAPDH antibody, ab8245, dilution 1:2,000) at 4 ℃ overnight and then washed three times in 1 × PBS-T. Horseradish peroxidase (HRP)-conjugated secondary goat anti-mouse antibody (Thermo Fisher Scientific, 31,430) and goat anti-rabbit (Thermo Fisher Scientific, 31,460) antibodies were diluted at 1:5.000 and added to the membranes for 2 h. Imaging was performed after washing PVDF membranes three times in 1 × PBS-T for 10 min using enhanced chemiluminescence and a horseradish peroxidase substrate (Super-Signal West Femto, Pierce, 34,094). Signals were detected using an Amersham Imager 680 machine (GE Life Sciences). GAPDH was used as a loading control while mitochondrial content was normalized to the mitochondrial protein TOM20.

Cryopreserved muscle samples were used to assess the respiratory chain activity. Respiratory chain complex measurements were performed as previously published [3].

### Long-range PCR for mitochondrial DNA deletions

The presence of mtDNA deletions in the major arc (10 kb) of the mitochondrial genome was assessed by long-range PCR. Custom-made primers were used (Fw: 5’-CCCTCTCTCCTACTCCTG; Rev: 5’-CAGGTGGTCAAGTATTTATGG). PCR amplification was performed on a T100 Thermal Cycler (Bio-Rad) using 1 ml of DNA and PrimeSTAR GXL DNA Polymerase (Takara Bio Europe) according to the manufacturer’s recommendations. PCR products were electrophoresed on a 0.7% agarose gel for 65 min. Three healthy and age-matched controls were included in the analysis.

### RNA sequencing

Bulk RNA sequencing was performed on frozen muscle biopsy specimens, as previously described [17]. In short, RNA was extracted from fresh-frozen biopsies using TRIzol (Invitrogen) and quantified using NanoDrop. Libraries for bulk RNA sequencing were prepared using NEBNext Poly(A) mRNA Magnetic Isolation Module and Ultra II Directional RNA Library Prep Kit for Illumina (New England BioLabs, cat. #E7490 and #E7760). The input RNA and the resulting libraries were analyzed with Agilent 4200 Tapestation for quality assessment. The libraries were sequenced using the NextSeq 550 and the NovaSeq 6000 Illumina platforms.

Histologically normal muscle biopsies were included as controls. These samples were obtained from the Johns Hopkins Neuromuscular Pathology Laboratory (*n* = 12), the Skeletal Muscle Biobank of the University of Kentucky (*n* = 8), and the National Institutes of Health (*n* = 13). The normal muscle biopsies from the Johns Hopkins Neuromuscular Pathology Laboratory were obtained for clinical purposes, but did not show any histological abnormality. The rest of the normal biopsies were obtained from healthy volunteers.

### Quantification and statistical analysis

Categorical variables are reported as numbers and percentages and were compared using Fisher’s exact test. Quantitative variables are reported as mean (± SD) and compared using Mann–Whitney or Kruskal–Wallis with Dunn’s multiple comparison tests. Spearman rank-order correlations were used to calculate the association’s quantitative variables. A *p* value < 0.05 was considered significant. GraphPad Prism 9.2.0 (GraphPad Software, Inc., La Jolla, CA, USA) was used for analysis.

## Results

### Exploratory patient cohort for molecular studies

Frozen biomaterial obtained from muscle biopsy samples was available for proteomic workup from seven out of eight DM2 patients (four female, three male). Muscle biopsies were collected for diagnostic purposes before genetic testing on these patients. Clinical data of the exploratory patient cohort are summarized in Table 1. The mean age at biopsy was 58 (SD ± 10.2) years. Of note, 6/8 patients showed mild to moderate CK elevation (ranging from 300 to 800 U/l; upper limit of normal (ULN): 190 U/l), while a positive family history was only reported by 2/7 patients, and the prevalence of diabetes was low in this cohort.

**Table 1:** Patient characteristics

Patient	Age at diagnosis	Sex	Family history	Muscle weakness	Myalgia	CK at biopsy (U/l)	Cataract	Diabetes
I*	52	f	No	Yes	Yes	536	Yes	No
II*	56	f	Yes	Yes	Yes	610	No	No
III*	54	m	No	Yes	No	560	Yes	No
IV*	68	f	No	Yes	Yes	732	Yes	Yes
V*	76	m	No	Yes	Yes	705	Yes	No
VI*	56	f	No	Yes	No	n/a	Yes	No
VII*	70	m	No	Yes	Yes	680	No	Yes
VIII	59	m	Yes	Yes	Yes	178	Yes	No

*CK* creatine kinase (units/liter); upper limit of normal 190 U/l

^*^Biopsies included in the proteomic analysis

### Evidence for mitochondrial dysfunction on the proteomic and transcriptomic level

Whole protein extracts of seven muscle biopsies underwent proteomic analysis as described above. In total, *n* = 3282 proteins were detected in the samples, of which *n* = 128 were upregulated and *n* = 62 were downregulated in the DM2 muscle biopsy samples compared to NDC. Strikingly, 16 of 62 downregulated proteins were involved in the mitochondrial oxidative phosphorylation (OXPHOS) system (see Fig. 1b). Four out of the ten most downregulated proteins (mt-CO1, mt-ND5, mt-CYB, SLC41A3) showed a mitochondrial localization, with many of them belonging to complex I, III, and IV of the respiratory chain. Interestingly, we found that a high number of downregulated proteins were encoded by mitochondrial genes (i.e., encoded on the mtDNA: mt-CO1, mt-ND5, and mt-CYB; see Fig. 1a, b, 5d) with the STRING analysis of differentially downregulated mitochondrial proteins illustrating the strong molecular interaction network between these dysregulated proteins. Notably, proteins associated with the large and exclusively nuclear-encoded respiratory chain complex II were not detected among the downregulated proteins. Furthermore, we detected a significant downregulation of translational activator of cytochrome oxidase subunit I (TACO1). TACO1 is required for translation of the COX-I subunit of complex IV, and TACO1 deficiency results in a complex IV defect, which may, in turn cause Leigh syndrome [15, 22]. Among the 46 downregulated proteins not directly linked to mitochondrial function, we found contractile (e.g., MYH13, MYH8, MYO1B), membrane-associated (e.g., CAV3, CACNG1), and RNA-binding proteins (e.g., LSM2). Conversely, only 2 of the 128 upregulated proteins were linked to mitochondrial function (UQCRH; OXCT1; see Fig. 1c). A compensatory upregulation of alternative, energy-producing cellular pathways (e.g., ß-oxidation, glycolysis, anaerobic pathways) was not evident on the proteomic level. Consistent with these results, GO-term analysis of biological processes indicated a strong downregulation of aerobic respiration, mitochondrial electron transport, and related processes (Supplemental Fig. 2). On the other hand, we observed an upregulation of processes related to cytoskeletal organization, unfolded protein response, and cell death (Supplemental Fig. 2).

We intersected the proteomic with the RNA sequencing data with a focus on differentially abundant mitochondrial proteins and their respective RNA transcripts. Here, we noticed a differential expression and uniform downregulation of the RNA transcripts coding for 13 out 16 downregulated mitochondrial proteins (Supplemental Fig. 3). Only 3 out of 16 transcripts were not significantly differentially expressed (UQCR10, COX7A1, PPTC7). Of note, the levels of wild type CNBP transcripts were also significantly lower in DM2 muscle biopsy samples.

### Light microscopy studies reveal structural abnormalities of mitochondria

**Fig. 2:**
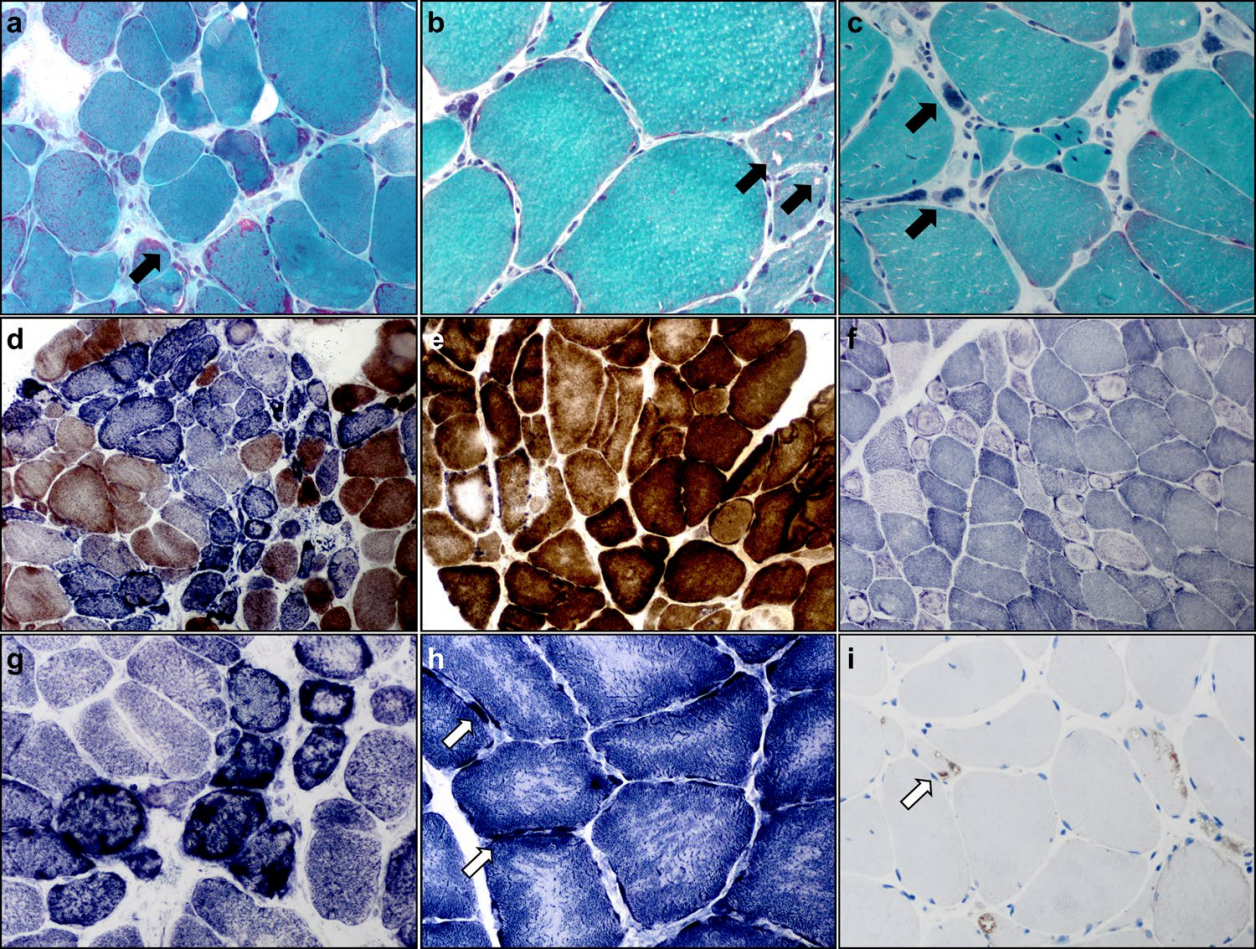
Histomorphological alterations of skeletal muscles from DM2 patients. Photomicrographs **a**–**i** illustrate histological findings in individual patients with DM2. **a**–**c** (Gömöri-trichrome staining): ragged red and split fibers (black arrow) (**a**; × 400), rimmed (black arrow) and non-rimmed vacuoles (**b**; × 400), grape-like nuclear clumps/bags (black arrow) (**c**; × 400). **d**, **e** (COX–SDH enzyme histochemical preparation): abundant and focally clustered COX-negative SDH-positive fibers (**d**; × 200), central displacement and variable irregularity of COX-activity in some fibers (**e**; × 400). **f** (NADH-TR staining): many myofibers showing ‘ringbinden’ and ring fibers mostly in type 2 fibers, while dark type 1 fibers only rarely show them (× 200). **g**–**h** (SDH staining): focally clustered ragged blue fibers (**g**, × 400), targetoid-like lesions in fibers with subsarcolemmal substrate accumulation (arrows) (**h**; × 600). **i** (p62 immunohistochemistry): autophagolysosomal aggregates (arrow) (× 400)

In addition to the exploratory DM2 cohort (*n* = 8), an independent set (*n* = 40) of muscle biopsy samples was available for confirmatory histological evaluation. All studied samples showed a combination of mild to severe fiber-size variation, an increased number of internalized myonuclei in hypertrophic and rounded fibers, abundant large pyknotic nuclear clumps with grape-like appearance (Fig. 1a, c, f), and, in some cases, ringbinden in ring fibers (Fig. 2f, Supplemental Fig. 3c). In more severe cases, endomysial connective tissue, including fat cell replacement of myofibers, was evident in Gömöri trichrome and EvG stains (not shown). ATPase preparations showed type-2 fiber atrophy. Ragged red or ragged blue and brown fibers were occasionally seen and sometimes abundantly clustering in some regions of the biopsies (Fig. 2g). Rimmed and non-rimmed vacuoles in the sarcoplasm of some fibers, myophaghocytosis, and necrotic fibers were present occasionally (Fig. 2b). A considerable number of vacuoles showed autophagolysosomal aggregates by p62 and LC3 stains (Fig. 2i). The nNOS stains and nonspecific esterase preparations did not highlight denervated fibers, while many of the large nuclear bags were nNOS negative (not shown). COX–SDH and SDH enzyme histochemistry revealed variably sized cap-like subsarcolemmal reaction product accumulating in all biopsies from the exploratory cohort (Fig. 2d, e). An age-inappropriate number of COX-negative fibers was present in all samples in the exploratory cohort, with COX-deficient fibers and coarse granular SDH positivity (Fig. 2d, g, h). In a few samples, we identified features of severe mitochondrial damage with many COX-negative/SDH-positive and ragged blue fibers (Fig. 2d). NADH-TR enzyme histochemistry to visualize the sarcotubular system and distribution of mitochondria (Fig. 2f) highlighted the subsarcolemmal accumulation of mitochondria and severe, often bizarre disruption of the sarcotubular system with coarseness, core-targetoid defects, ring fibers, and rubbed-out lesions in the biopsy samples at varying degrees of severity. In the confirmatory cohort, in addition to routine stains, at least one additional mitochondrial enzyme histochemical preparation (SDH, COX–SDH, COX or NADH-TR) was available for each biopsy. All patients (40/40) showed one or more of the above-described features indicative of mitochondrial dysfunction (subsarcolemmal caps, coarse granular SDH positivity, COX negativity of some fibers, and the above age-related normal values).

### Widespread ultrastructural abnormalities of mitochondria and additional features

**Fig. 3:**
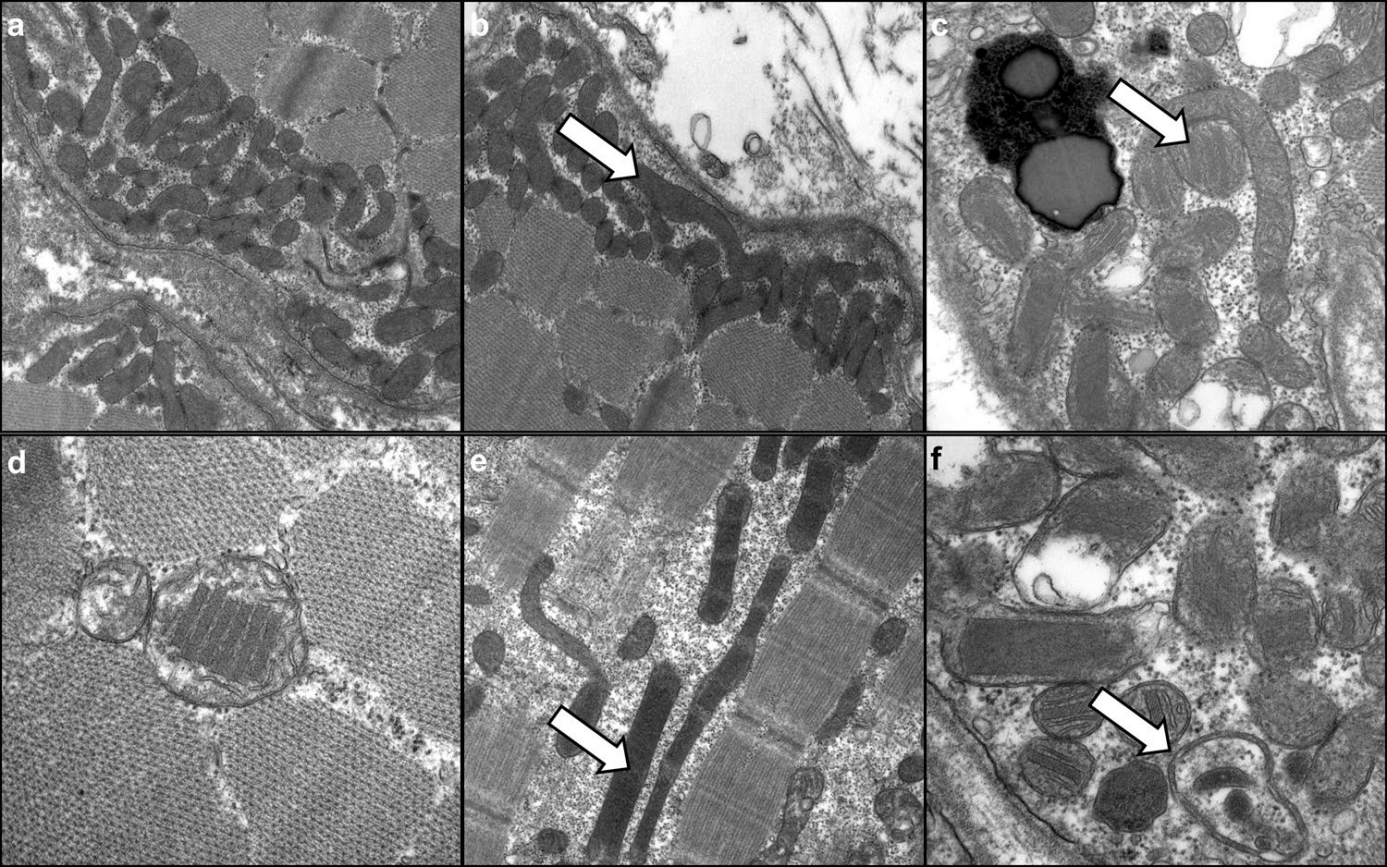
Spectrum of ultrastructural features of abnormal mitochondria in DM2. **a** Subsarcolemmal accumulation of swollen mitochondria (× 20,000). **b** Subsarcolemmal accumulation of swollen mitochondria, including dysmorphic, ‘worm-shaped’ elongated mitochondria (arrow) (× 15,000). **c** Dysmorphic, ‘worm-shaped’ elongated mitochondria with type I paracrystalline inclusions (arrow) (× 30,000). **d** Pathological mitochondria with type I paracrystalline inclusions (× 50,000). **e** Pathological, elongated mitochondria with type II paracrystalline inclusions (arrow) (× 20,000). **f** Pathological mitochondria, including one with circular cristae (arrow) and several paracrystalline inclusions type I and II (× 50,000)

On transmission electron microscopy studies, mitochondria showed striking subsarcolemmal accumulation in many myofibers (Fig. 3a, b). They often appeared enlarged with packed cristae and elongated (Fig. 3b, c), sometimes including paracrystalline inclusions (PCI) of type I and II (Fig. 3c, e) with variable architecture (Fig. 3f), reflecting profound mitochondrial dysfunction and the overt disruption of regular mitochondrial placement in muscle cells evident at light microscopic levels. All skeletal muscle samples showed mitochondrial abnormalities with quantitative rather than qualitative differences between the individual cases. Quantification of 667 mitochondria in 13 photomicrographs from individual patients at 12.000-fold magnification revealed ultrastructural abnormalities in 49% of mitochondria (type 1 PCI in 7.4%, type 2 PCI in 4.8%, circular cristae in 2.8%, swollen and enlarged mitochondria in 34%).

Additional features included I-band–Z-band–I-band (I-Z-I)-like structures in atrophic fibers (Supplemental Fig. 4a,d), nuclear clumps (Supplemental Fig. 4b), ring fibers and ringbinden (Supplemental Fig. 4c), filamentous bodies (Supplemental Fig. 4e), and zebra bodies (Supplemental Fig. 4f).

### Evidence for impaired auto-/and mitophagic flux

**Fig. 4:**
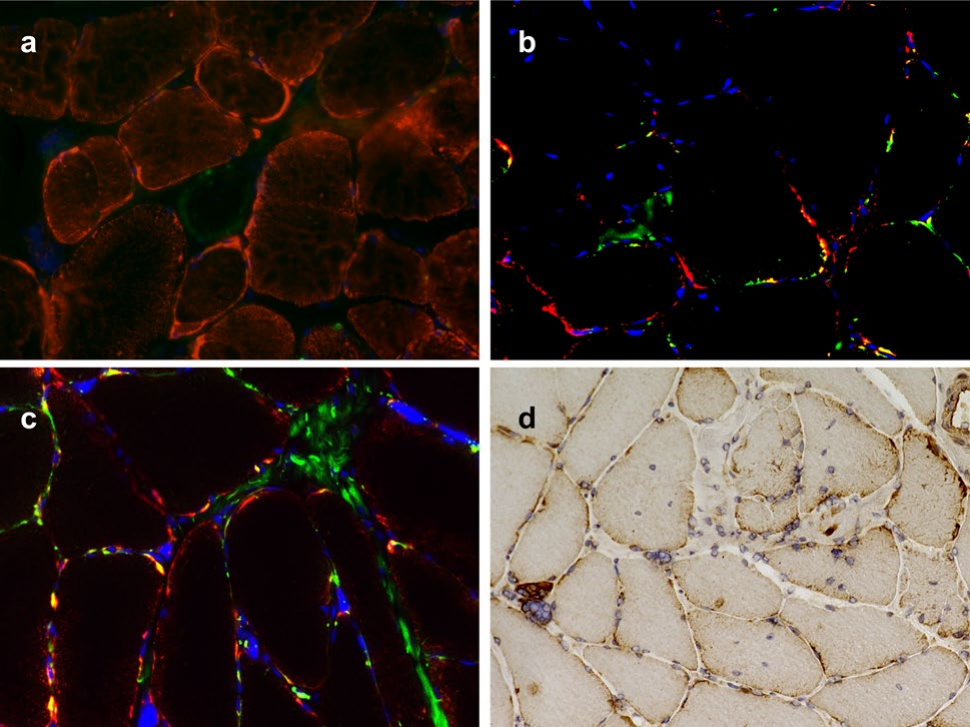
Auto- and mitophagy in DM2 muscle biopsy samples. **a**–**c** Representative immunofluorescence of individual biopsy samples derived from DM2 patients with co-localization of mitochondrial marker protein TOM20 (**a**; red) with LC-3 (green); TOM20 (**b**; red) with p62 (green); and COX-IV (c; red) with p62 (green). DAPI staining of nuclei in blue (**b**, **c**; magnification × 400). While subsarcolemmal accumulated mitochondria are visible by immunofluorescence, co-staining (yellow) is only evident for p62 and TOM20/COX-IV (**b**, **c**). **d** Immunohistochemistry for the mitophagy marker BNIP3 shows a strong staining reaction in the subsarcolemmal region in many myofibers (× 200)

As widespread mitochondrial abnormalities were evident at the histopathologic, ultrastructural, proteomic, and transcriptomic levels, we performed additional double immunofluorescence and immunohistochemistry to study auto- and mitophagic processes possibly triggered by these events. Immunofluorescence using mitochondrial markers TOM20 and COX-IV and autophagy markers LC-3 (Fig. 4a) and p62 (Fig. 4b, c) again showed a subsarcolemmal accumulation of mitochondria. Subsarcolemmaly accumulated mitochondria co-localized with p62 but not with LC-3. Immunohistochemistry for the mitophagy marker BNIP3 showed a positive staining reaction in the subsarcolemmal region in many fibers (Fig. 4d).

### Respiratory chain immunoblotting and enzymology, mtDNA copy number and deletion analyses

We performed immunoblotting of the respiratory chain complexes I-V and mtDNA copy number determination in six out of seven muscle biopsy samples that also underwent mass spectrometry-based proteomic analysis. Three healthy and age-matched controls were included and processed for immunoblotting on the same PVDF membrane.

**Fig. 5:**
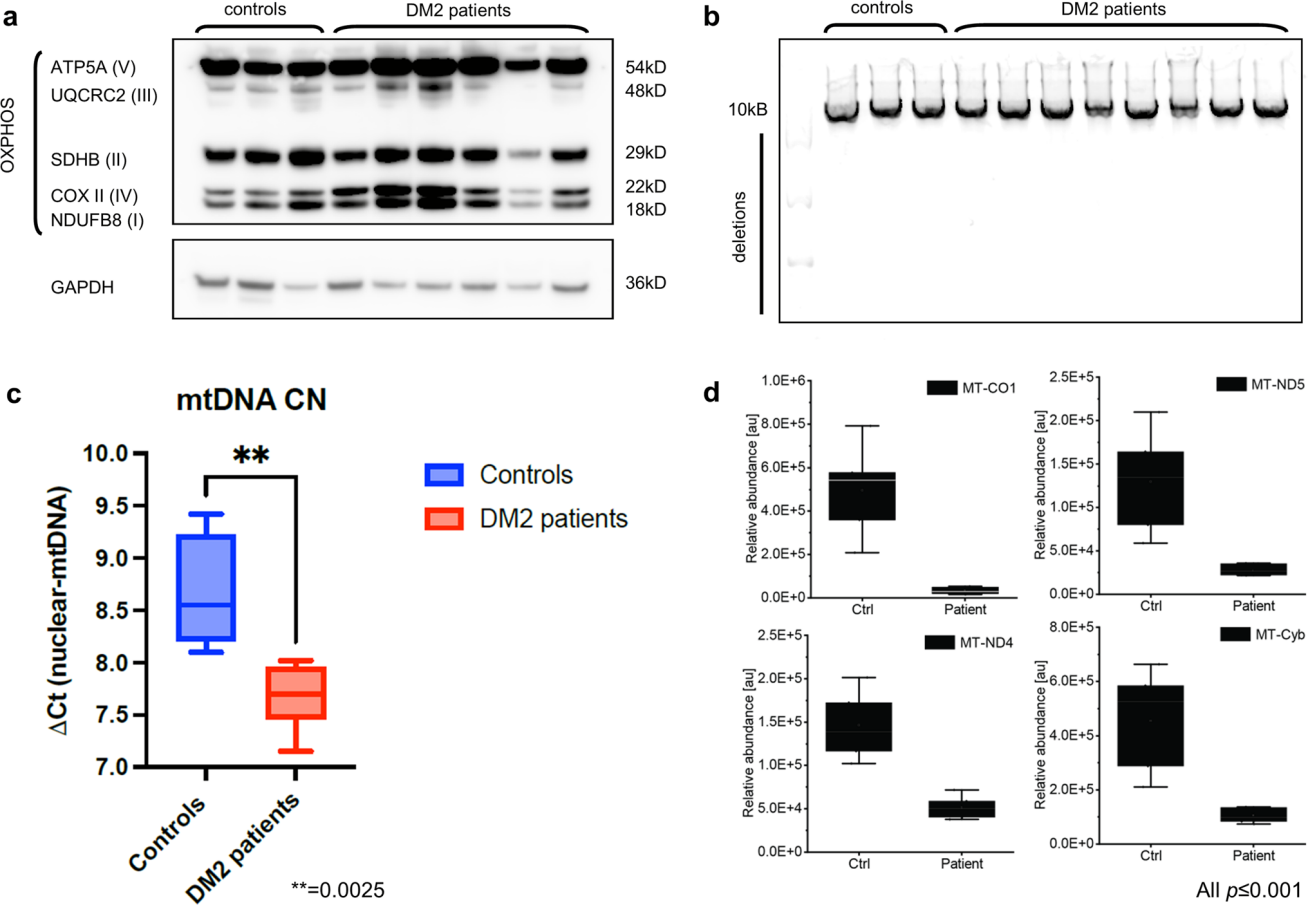
OXPHOS immunoblotting, long-range PCR, mtDNA copy number analysis and proteomic analysis of mtDNA-encoded OXPHOS subunits. **a** Respiratory chain subcomplexes western blot total of cell lysate of skeletal muscle biopsy samples derived from control and DM2 patients. OXPHOS complexes are not differentially affected in controls and patients. **b** Long-range PCR of the major arc (10kB) of the human mitochondrial genome. No mtDNA subfragments (additional bands) indicative of mtDNA deletions were detected in patients and controls. **c** PCR-based mtDNA copy number analysis in DM2 and control samples showing a reduced copy number in the patient group. The averages of both groups are represented as horizontal lines, and graphs show mean ± SD and interquartile range. **d** Boxplots of mitochondrial-encoded proteins mt-CO1, mt-ND-5, mt-ND, and MT-CYB showing a significant downregulation on the proteomic level in DM2 patients vs NDC (graphs show mean ± SD and interquartile range)

Western blotting analysis showed normal levels of mitochondrial proteins (Complex I: NDUFB8; Complex II: SDHB; Complex III: UQCRC2; Complex IV: MTCO1; Complex V: ATP5A) in control-derived muscle (*n* = 3) and patient-derived muscle (*n* = 6) (Fig. 5a). Quantitative analysis (densitometry) of protein levels did not show statistically significant differences between patient- and control-derived samples. GAPDH was used as a loading control, and the quantitative analysis was done based on the mitochondrial marker protein TOM20.

Respiratory chain enzymology showed a trend toward a combined reduction in complex I (NADH/Coenzyme Q10) and IV (COX) activities (e.g., in patients V and VII), while complex II/III and V activities were not affected (Supplemental Table 1).

PCR-based mtDNA copy number measurements were performed on six muscle biopsy specimens. The mtDNA-CN measurement showed a statistically significant reduction in mtDNA-CN in DM2 patients compared to healthy controls (Fig. 5c).

To further investigate a possible link between mitochondrial dysfunction and deletions of the mitochondrial DNA, we performed a long-range PCR of the 10 kb (major) arc of the mtDNA as the majority of mtDNA deletions are known to be located in the major arc [2]. Using this technique, we did not detect age-inappropriate mtDNA deletions in the samples derived from DM2 patients (Fig. 5b).

## Discussion

In this study, we investigated the pathophysiological underpinnings of myotonic dystrophy type 2 using a combined histological, ultrastructural, molecular, proteomic, and transcriptomic approach, including validation of the morphological and ultrastructural findings in an independent large cohort of DM2 patients. Mitochondrial dysfunction was evident on the morphological and proteomic level, and we detected significantly reduced mtDNA copy numbers, raising the possibility of an mtDNA maintenance defect. The intersection of proteomic and transcriptomic data revealed a concordant downregulation of RNA transcript and protein levels of subunits of respiratory chain complexes I, III, and IV and a complex IV translation factor (TACO1). Respiratory chain enzymology showed a trend toward the reduction of complexes I and IV. Moreover, immunofluorescence and immunohistochemistry focusing on auto- and mitophagy showed evidence of impaired mitophagy and mitophagosome formation. In summary, mitochondrial dysfunction in DM2 may be explained by altered RNA expression, subsequent dysregulation of key mitochondrial proteins and impaired clearance of damaged mitochondria.

State-of-the-art proteomic techniques have not been used thus far to discover pathomechanisms in DM2 muscle biopsy specimens. A previous study performed two-dimensional gel electrophoresis and mass spectrometry in DM2 myotubes [20]. The authors reported alterations in proteasomal protein processing pathways (e.g., PSA6, PSD13) and mitochondrial metabolism (e.g., HSP60, GRP75, EFTU) without exploring these findings in more depth or validating the findings in muscle biopsy samples. In contrast to our results, the authors did not detect a reduction in proteins associated with complexes I, III, and IV, possibly due to using a less sensitive and in-between outdated proteomic method.

In DM1, however, mitochondrial abnormalities, including impaired mitochondrial biogenesis, have been reported and even proposed as potential therapeutic targets, e.g., treatable with metformin [4, 6]. Interestingly, one study also described decreased OPA1, MFN2, DRP1, and PARKIN levels in DM1-derived fibroblasts, pointing toward ineffective mitophagic mechanisms [4]. Moreover, abnormal mitochondrial structures had been described in DM1 and linked to the expression of aberrant *DMPK* isoforms [16] with the overexpression of *hDMPK A,* resulting in mitochondrial fragmentation and clustering in the perinuclear regions. Recently, a transcriptome analysis of DM1 myoblasts revealed altered pathways linked to mitochondria, e.g., protein insertion into mitochondrial membrane involved in apoptotic signal pathways, regulation of mitochondrial depolarization, ATP hydrolysis-coupled proton transport, and the oxidation–reduction process [24].

Interestingly, mitochondrial dysfunction has also been reported as a prominent feature of oculopharyngeal muscular dystrophy (OPMD), another repeat expansion disease of the skeletal muscle [1, 27]. In the context of OPMD, it has been hypothesized that aberrant and premature degradation of RNA (e.g., RNA encoding mitochondrial proteins) may cause mitochondrial dysfunction [1].

Aberrant splicing, the misprocessing of RNA and proteins, and sequestration of transcription and splice modification molecules (e.g., muscleblind-like proteins (MBNL)) are key players in the pathophysiology of DM2. For this reason, similar mechanisms, e.g., mis-splicing, premature degradation and subsequent alterations in wild type RNA levels, could be hypothesized to be involved in developing mitochondrial dysfunction in DM2. Indeed, our transcriptomic analysis revealed reduced abundances of mRNA transcripts related to mitochondrial function (e.g., mt-CO1, mt-ND5, mt-CYB), with a subsequent downregulation on the protein level. Thus, reduced expression and/or premature degradation of wild type mRNA may be considered the most upstream event in the pathophysiology of mitochondrial dysfunction in the context of DM2.

Interestingly, genetic defects in mitochondrial tRNA synthetases have previously been linked to DM-like phenotypes and complex IV deficiency [9]. These proteins would, in theory, be vulnerable to the above-mentioned mis-splicing mechanisms known to be involved in the pathophysiology of DM2. Still, we did not detect dysregulation of tRNA-related proteins in our dataset. We did, however, detect a downregulation of the protein TACO1, which represents a critical translation factor for the complex IV subunit COX-I. While cellular TACO1 decrease could be explained by misprocessing or premature degradation of the corresponding RNA, TACO1 deficiency has previously been linked to cytochrome c/complex IV deficiency and mitochondrial disease [26]. Thus, TACO1 deficiency could partly explain the excess COX-negative fibers seen in DM2. Additional molecular studies are needed to explore the underlying mechanisms further.

While we observed widespread mitochondrial abnormalities on the electron microscopic level, there were no signs of compensatory mitophagy, as presumably expected in severe mitochondrial disorders. In our cohort, LC-3 and p62 positive intracellular structures were visible, in some cases with pronounced subsarcolemmal staining at sites of mitochondrial accumulation. Double immunofluorescence of LC-3 and p62 with COX-IV and TOM20 did indicate a subarcolemmal accumulation of mitochondria, thus confirming light microscopic and ultrastructural findings. However, co-localization of mitochondrial markers TOM20 and COX-IV was only evident with p62 and not LC-3. In addition, we studied the expression of mitophagy marker BNIP3 by immunohistochemistry. While p62 and BNIP3 label damaged mitochondria for degradation and serve as adapter proteins, LC-3 is localized at the autophagosome membranes and is considered a key player in mitophagy [12, 28]. This data indicates a disturbed autophagic flux resulting in impaired LC-3 positive mitophagosome formation. This could lead to defective mitophagy and subsequent accumulation of abnormal mitochondria. To our knowledge, this is the first data hinting toward impaired mitophagy in the context of DM2. Interestingly, a recent study has evoked dysfunctional mito- and autophagy in DM1, pointing to the possibility of shared pathophysiologic mechanisms at play in DM1 and DM2 [19].

Although we detected reduced mtDNA copy number, we could not identify the accumulation of muscle-specific mtDNA deletions, as reported in DM1 [21], raising the possibility that underlying mitochondrial changes may have different mechanisms compared to DM1. While we observed a trend toward reduced complex IV (mtCO1) by immunoblotting, we did not detect any statistically significant downregulation of OXPHOS complexes overall. This may be explained by different antigens/antibodies used as targets for immunoblotting (e.g., for complexes I and III) and the sensitivity of the method used on whole muscle extracts with varying degrees of mitochondrial damage. However, our proteomic results were validated by IHC staining (e.g., COX staining for complex IV activity, NADH-TR for complex I, SDH for complex II) and ultrastructural studies indicating mitochondrial pathology.

While our data point toward a significant role of mitochondrial pathology in DM2, further studies are needed to corroborate these findings and assess the functional impact of mitochondrial dysfunction on cellular metabolism. The latter could be studied using a Seahorse assay, an established method for determining cellular energy metabolism [7]. Seahorse assays have been successfully used on fibroblasts in the context of DM1 [4].

However, several methodological questions need to be addressed, e.g., identifying a valid cell culture model recapitulating DM2 pathophysiology. Along this line, it remains to be shown whether the peculiar DM2 (mitochondrial) pathology can be recapitulated in cell culture models, e.g., myoblasts. It may be essential to use differentiated cells, e.g., myotubes, and even then, the model’s validity must be demonstrated conclusively. In addition, transcriptomic approaches may help study the DM2 pathophysiology on the RNA level, including the analysis of alternative splicing in large datasets.

## Conclusions

Myotonic dystrophy type 2 is a progressive and currently incurable multisystemic disease, for which elucidation of underlying pathomechanisms and subsequent development of novel molecular treatment targets are urgently needed. In this study, we performed multi-level disease profiling revealing mitochondrial dysfunction with pronounced involvement of complex I, III, and IV and associated translation factors. While DM2 is not a primary mitochondrial disease per se, we believe that mitochondria are involved in its pathophysiology and, thus, clinical manifestation. If the functional impact of these abnormalities can be conclusively demonstrated, e.g., by functional assays from cell culture, mitochondria might indeed emerge as novel additional treatment targets in DM2.

### Supplementary Information

Below is the link to the electronic supplementary material.

Supplementary file1 (DOCX 42 kb)

Supplementary file2 (DOCX 215 kb)

Supplementary file3 (DOCX 122 kb)

Supplementary file4 (DOCX 2810 kb)

Supplementary file5 (DOCX 13 kb)

## Data Availability

The histological and ultrastructural data supporting this study’s findings are available from the corresponding author, WS, upon reasonable request. The full proteomic dataset is available via ProteomeXchange with identifier PXD044286.
