# Supplementary material for: Multi-level profiling unravels mitochondrial dysfunction in myotonic dystrophy type 2
Source: Acta Neuropathol. 2024 Jan 19;147(1):19. doi: 10.1007/s00401-023-02673-y (PMC10799095; doi:10.1007/s00401-023-02673-y)
Supplement: Supplementary file 1 — Supplementary file1 (DOCX 42 kb) [file 401_2023_2673_MOESM1_ESM.docx]

**
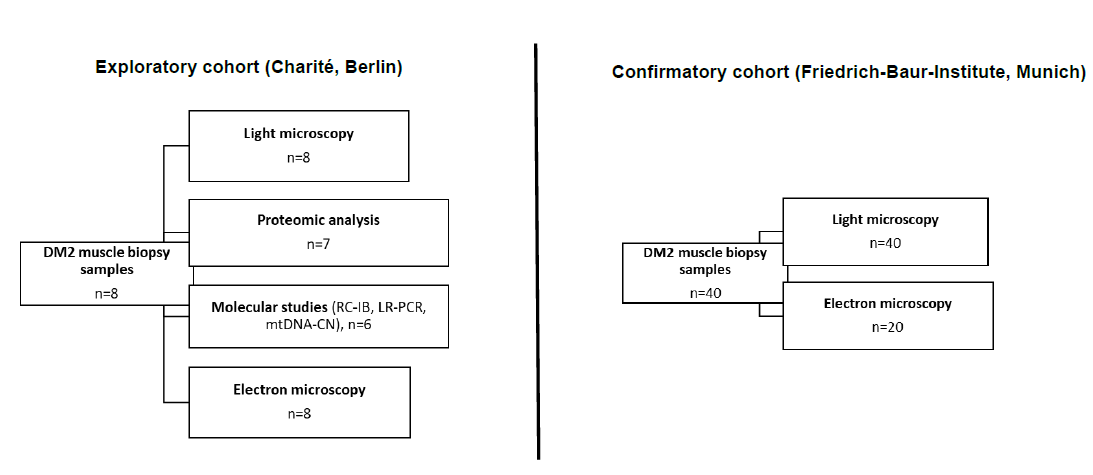
**

**Supplemental Fig 1** Overview of cohorts and workflow.

A cohort consisting of eight DM2 patients from Berlin, Germany, from which frozen biological material was available, served as exploratory cohort for molecular and morphological studies.

A separate patient cohort consisting of 40 DM2 biopsy samples from Munich, Germany, was used as independent confirmatory cohort for light and electron microscopy studies.
