# Supplementary material for: Multi-level profiling unravels mitochondrial dysfunction in myotonic dystrophy type 2
Source: Acta Neuropathol. 2024 Jan 19;147(1):19. doi: 10.1007/s00401-023-02673-y (PMC10799095; doi:10.1007/s00401-023-02673-y)
Supplement: Supplementary file 2 — Supplementary file2 (DOCX 215 kb) [file 401_2023_2673_MOESM2_ESM.docx]

**
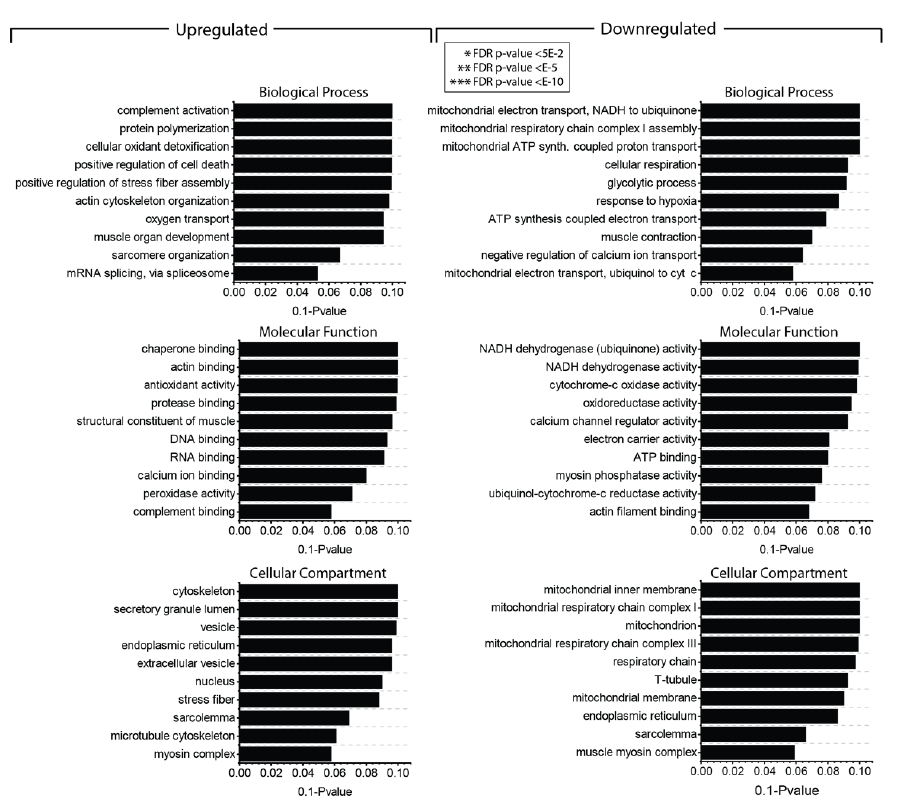
**

**Supplemental Fig 1** GO-Term analysis of differentially abundant proteins in DM2 muscle biopsy samples studied by unbiased proteomic analysis.
