# Supplementary material for: Multi-level profiling unravels mitochondrial dysfunction in myotonic dystrophy type 2
Source: Acta Neuropathol. 2024 Jan 19;147(1):19. doi: 10.1007/s00401-023-02673-y (PMC10799095; doi:10.1007/s00401-023-02673-y)
Supplement: Supplementary file 3 — Supplementary file3 (DOCX 122 kb) [file 401_2023_2673_MOESM3_ESM.docx]

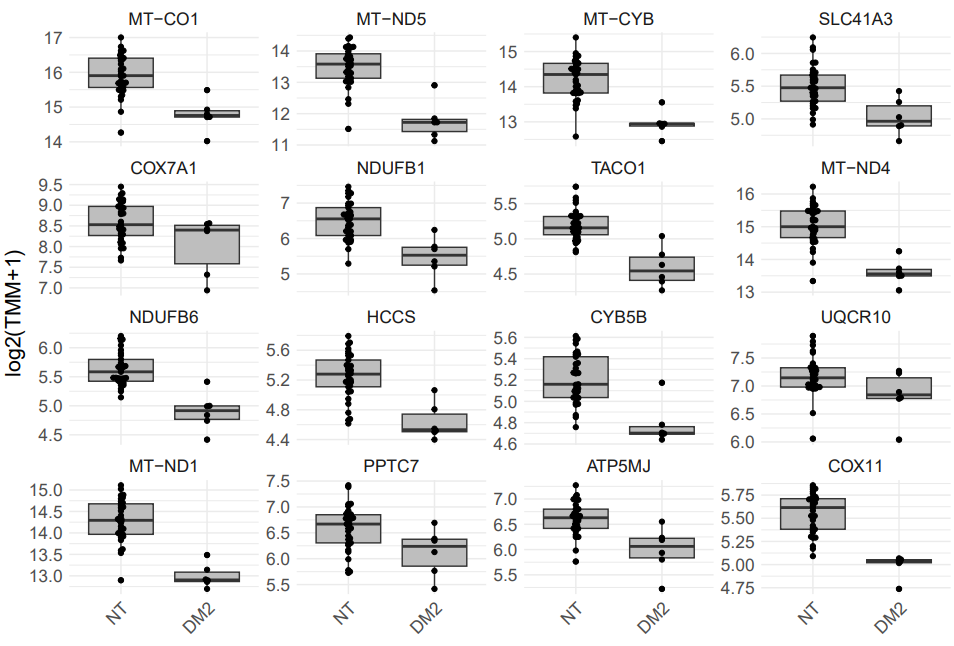


**Supplemental Fig 3** Graphs represent log-scaled normalized expression levels of differentially expressed mRNA of 16 mitochondria-associated proteins in DM2 vs. non-diseased controls (NT= histologically normal tissue) muscle biopsy samples. All transcripts except UQCR10, COX7A1, and PPTC7 are significantly differentially expressed in DM2 muscle (all *p*<0.02).
