# Supplementary material for: Multi-level profiling unravels mitochondrial dysfunction in myotonic dystrophy type 2
Source: Acta Neuropathol. 2024 Jan 19;147(1):19. doi: 10.1007/s00401-023-02673-y (PMC10799095; doi:10.1007/s00401-023-02673-y)
Supplement: Supplementary file 4 — Supplementary file4 (DOCX 2810 kb) [file 401_2023_2673_MOESM4_ESM.docx]

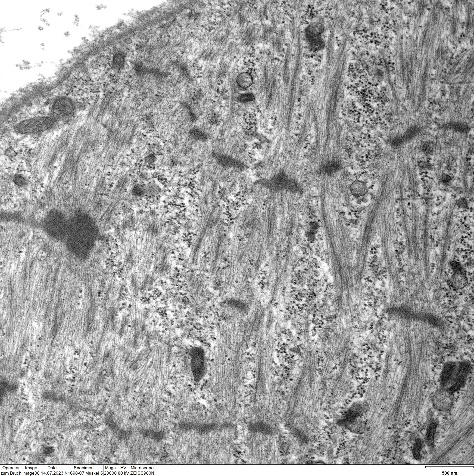

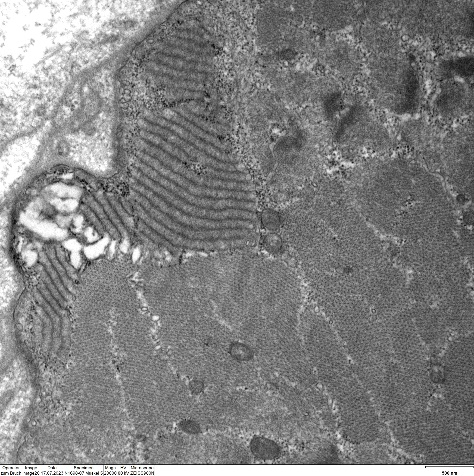

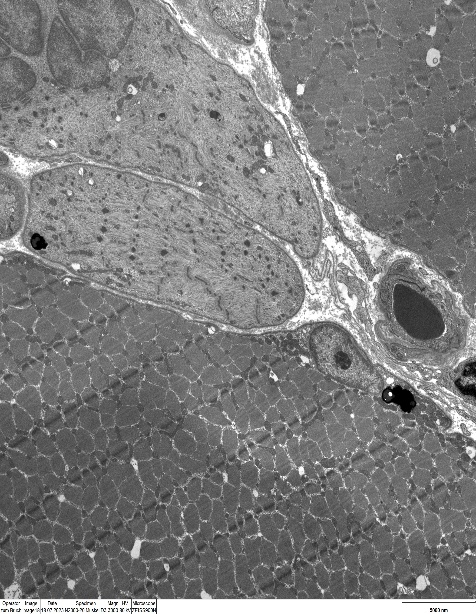

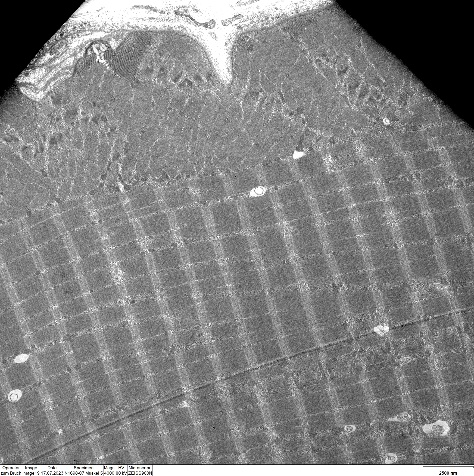

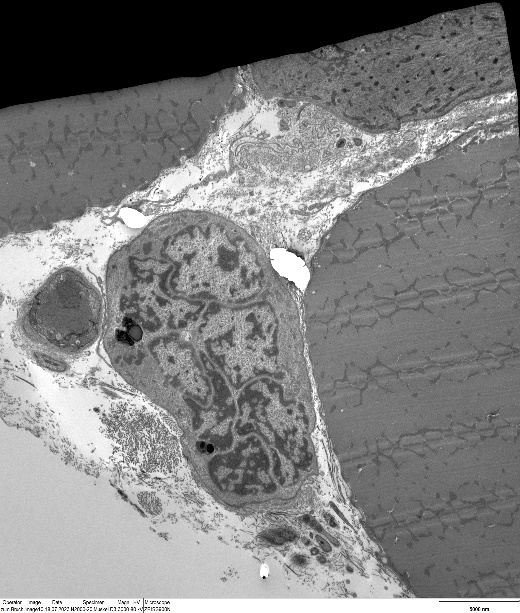

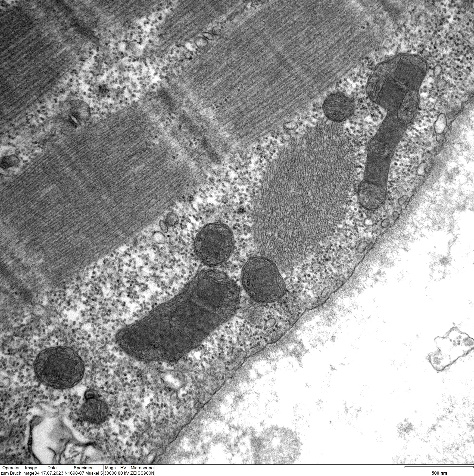


**a**

**b**

**c**

**d**

**e**

**f**

**Supplemental Fig 4** Ultrastructural features of Myotonic Dystrophy Type 2.

Photomicrographs a-f illustrate ultrastructural findings in three individual patients.

a: Two atrophic fibers including one with nuclear clumps (arrow) (x4,000).

b: Nuclear clumps (arrow) (x3,000).

c: Ring fiber with ringbinden (x4,000).

d: Severely damaged myofiber with remaining Z-bands to which sparse I-bands are attached (x20,000).

e: Filamentous aggregate, likely corresponding to a filamentous body in between mitochondria with paracrystalline inclusions (arrow) (x30,000).

f: Subsarcolemmal zebra-body (arrow) (x20,000).
