# Supplementary material for: Multi-level profiling unravels mitochondrial dysfunction in myotonic dystrophy type 2
Source: Acta Neuropathol. 2024 Jan 19;147(1):19. doi: 10.1007/s00401-023-02673-y (PMC10799095; doi:10.1007/s00401-023-02673-y)
Supplement: Supplementary file 5 — Supplementary file5 (DOCX 13 kb) [file 401_2023_2673_MOESM5_ESM.docx]

**Supplemental Table 1** Respiratory chain enzymology.

| **Patient** | NCP | NADH/ CoQ | Succinate Cytochrome c | COX | Citrate synthase (CS) | NADH/CS | Succinate/CS | COX/CS |
| --- | --- | --- | --- | --- | --- | --- | --- | --- |
| LLN |  | 15,8 | 6,0 | 112,0 | 45,0 | 0,17 | 0,08 | 1,10 |
| I | 2,9 | ND | 8,4 | 127,2 | 102,1 | ND | 0,08 | 1,25 |
| II | 4,5 | ND | 8,2 | 139,3 | 95,8 | ND | 0,09 | 1,45 |
| III | 4,2 | 3,4 | 10,7 | 150,2 | 92,9 | 0,04 | 0,12 | 1,62 |
| IV | 5,4 | ND | 6,1 | 81,4 | 54,8 | ND | 0,11 | 1,49 |
| V | 8,3 | 11,2 | 6,3 | 95,6 | 47,1 | 0,24 | 0,13 | 2,03 |
| VI | 7,6 | 39,5 | 17,3 | 233,2 | 92,5 | 0,43 | 0,19 | 2,52 |
| VII | 7,8 | 0,9 | 6 | 72,7 | 48,2 | 0,02 | 0,12 | 1,51 |
|  |  |  |  |  |  |  |  |  |
| DM1/I | 6,8 | 18,5 | 13,7 | 160,1 | 84,6 | 0,22 | 0,16 | 1,89 |
| DM1/II | 8,5 | 7,2 | 8 | 96,2 | 41,9 | 0,17 | 0,19 | 2,30 |
| DM1/III | 8,7 | 6,2 | 7,4 | 107,9 | 59,7 | 0,10 | 0,12 | 1,81 |

LLN: lower limit of normal; NCP: non-collagenous protein
